# Towards nanoprinting with metals on graphene

**DOI:** 10.1038/ncomms9071

**Published:** 2015-08-28

**Authors:** G. Melinte, S. Moldovan, C. Hirlimann, X. Liu, S. Bégin-Colin, D. Bégin, F. Banhart, C. Pham-Huu, O. Ersen

**Affiliations:** 1 Institut de Physique et Chimie des Matériaux de Strasbourg (IPCMS), UMR 7504 CNRS-Université de Strasbourg (UdS), 23, rue du Loess, 67034 Strasbourg cedex 2, France; 2 Institut de Chimie et Procédés pour l'Energie, l'Environnement et la Santé (ICPEES), UMR 7515 CNRS, ECPM, Université de Strasbourg (UdS), 25, rue Becquerel, 67087 Strasbourg, France

## Abstract

Graphene and carbon nanotubes are envisaged as suitable materials for the fabrication of the new generation of nanoelectronics. The controlled patterning of such nanostructures with metal nanoparticles is conditioned by the transfer between a recipient and the surface to pattern. Electromigration under the impact of an applied voltage stands at the base of printing discrete digits at the nanoscale. Here we report the use of carbon nanotubes as nanoreservoirs for iron nanoparticles transfer on few-layer graphene. An initial Joule-induced annealing is required to ensure the control of the mass transfer with the nanotube acting as a ‘pen' for the writing process. By applying a voltage, the tube filled with metal nanoparticles can deposit metal on the surface of the graphene sheet at precise locations. The reverse transfer of nanoparticles from the graphene surface to the nanotube when changing the voltage polarity opens the way for error corrections.

## Abstract (web summary)

The precise delivery of materials onto graphene is important for nano-processing but little is known about the mechanisms of such processes. Here, the authors use a range of microscopic techniques for the real-time observation of nanoparticle transfer from the inner channel of a carbon nanotube onto graphene.

## Graphical abstract

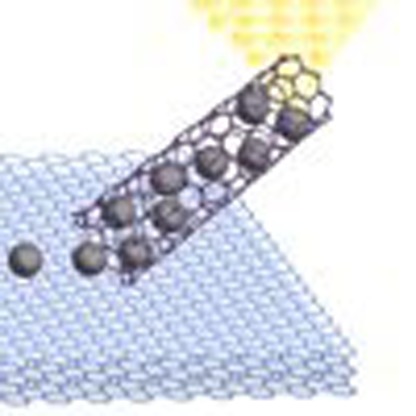

The physical properties of graphene^1^ and carbon nanotubes (CNTs)^2^ recommend them as promising candidates for the fabrication of nanodevices with applications in several fields. For instance, electrical interconnectors^3^, pipelines for femtogramme mass transport^4^,^5^,^6^,^7^,^8^ and nanoswitches^9^,^10^ can be assembled from CNTs filled with metallic nanoparticles. In addition, graphene flakes decorated with nanoparticles are gaining growing interest as alternative supports in the fields of catalysis^11^,^12^, energy storage^13^,^14^ and optoelectronics^15^.

The prospects of delivering mass onto predefined locations in the femtogramme regime^16^ or of generating nanowires^17^ in a tube plead for developing adapted approaches for the investigation of mass transport in or out of CNTs. In the past few years, significant progress was made in the understanding of the phenomena at the basis of mass transport on the inner^18^ and/or outer^19^ surface of CNTs. The pioneering work of Svensson *et al.*^20^ emphasizes the role of the electromigration force on the nanoparticle transfer from CNTs employed as ‘nanopipettes' on gold substrates. Today, however, only little is known on the mechanisms, conditions and constraints governing the process of materials transfer from one support to another under the impact of an electrical potential difference.

Here, we focus on the possibility of using CNTs filled with metal nanoparticles as a ‘pen' and a few-layer graphene (FLG) flake as a ‘paper board'. By combining high-resolution transmission electron microscopy (HR-TEM) and scanning tunnelling microscopy (STM) via a specific sample holder^21^, we are able to carry out the real-time observation of a nanoparticle (NP) transfer from the inner channel of a CNT onto FLG flakes. This approach can lay down the scientific ground work necessary for the development of nanoprinting techniques. The study takes advantage of the electron energy loss spectroscopy (EELS) technique for monitoring the chemical changes that occur during nanoparticles transfer while the use of *in situ* TEM–STM allows for the observation of the NPs transfer process from a mechanistic perspective. More specific, one aims monitoring the NPs motion from the CNT onto the FLG surface and vice versa. The application of electrical pulses induces high crystalline and structural stability to the tube, which is a mandatory prerequisite for performing electrically controlled nanoparticle transfer. The impact of different factors controlling the nanoparticles transfer (nanotubes structure, iron nanoparticles properties) is highlighted together with the migration of nanoparticles on a graphene substrate through an Ostwald ripening-like process, as methodology to create a complete platform for nanoparticles printing.

## Results

### Joule activation of carbon nanotubes conductivity

**Figure 1:**
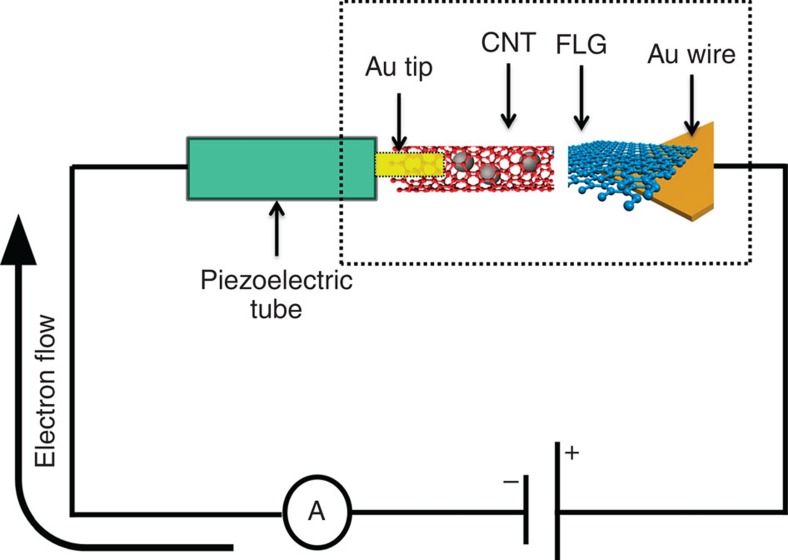
Sketch of the TEM–STM experimental setup. The setup used for the Joule activation of the CNTs' conductivity, the nanoparticle transfer and manipulation on the FLG flakes.

The experimental setup consists of a Fe_3−*x*_O_4_-filled CNT which is mounted on a sharp Au tip, on the one side, and on the other side an Au wire (0.3 mm in diameter) supporting a FLG sheet. The schematic drawing of the experimental setup is presented in Fig. 1. For a detailed description of the experiment preparation see the Methods section. The use of CNTs as pipelines for material transport requires a high degree of filling with metal nanoparticles. The commercially available multi-walled CNTs with a large diameter appear as a natural choice, as they exhibit diameters between 35 and 50 nm ensuring large filling volumes.

**Figure 2:**
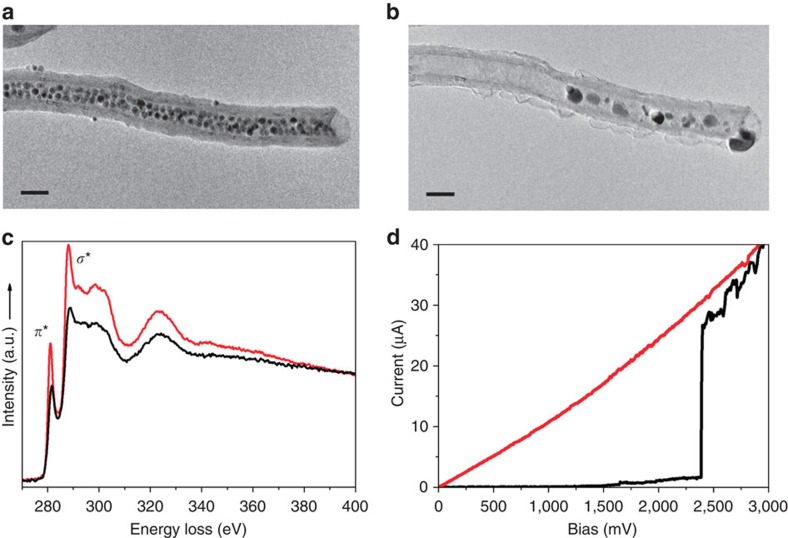
CNT filled with Fe-based nanoparticles prior and after application of a bias voltage. (**a**) Fe_3−*x*_O_4_ NPs-filled CNT (before) and (**b**) metallic Fe/Fe_*x*_C_*y*_ NPs-filled CNT (after). (**c**) EELS spectra of the CNT before (black line) and after (red line) a voltage pulse. (**d**) Characteristics *I*(*V*) curves of the raw Fe_3−*x*_O_4_ NPs-filled CNTs (black line) and the Joule annealed CNTs (red line). Scale bars, 50 nm.

A typical TEM micrograph of the Fe_3−*x*_O_4_-filled CNT is shown on Fig. 2a. The HR-TEM micrographs (Supplementary Fig. 1) show that the CNT external walls contain a large amount of residual amorphous carbon. However, the well-defined π* and σ* features in the EELS spectra (Fig. 2c, black line) do identify a sp^2^-like network. The residual amorphous carbon within a CNT and the structural defects in the graphitic layers are expected to impact the tube behaviour when applying an electric potential. Indeed, when a bias voltage is applied to the CNT for the first time, the short (100 ms) current pulse abruptly changes the CNT's structure (Fig. 2b). At the same time, one can observe the graphitization of the amorphous carbon coating layer^22^ as induced by the Joule annealing of the nanotube. This effect is proved by the EELS spectra (Fig. 2c, red line), which shows the enhancing of the π* and σ* signatures of the graphitic structure with respect to the initial spectra. The characteristic *I*(*V*) curve as recorded on a raw Fe_3−*x*_O_4_-filled CNT (Fig. 2d, black line) is characterized by a significant jump in the current intensity in the voltage interval of 2–2.5 V. This backs up the occurrence of a carbon graphitization in the walls as identified by HR-TEM and EELS. Due to the fast recording of the *I*(*V*) curve (100 ms) in our experimental conditions, the current outburst appears to be instant. However, when the bias is varied at a smaller rate, the current increase appears in a larger voltage interval. The nearly linear profile of the *I*(*V*) curve acquired in a second run (Fig. 2d, red line) demonstrates that the NPs/CNT system is converted to an Ohmic device.

Moreover, the encapsulated NPs undergo a fast thermal or electrochemical reduction and a subsequent coalescence phenomenon, induced by the large thermal energy injected into the CNT via the Joule heating. The EELS spectrum displayed in Supplementary Fig. 2a (red line) shows that after applying the voltage pulse, the O-K edge (∼530 eV) significantly weakens as compared with the initial spectrum of the Fe_3−*x*_O_4_-filled CNT (black line). Furthermore, the Fe L_3_:Fe L_2_ intensity ratio decreases from ∼4.3 to ∼2.8, before and after the *I*(*V*) curve recording, a phenomenon related to the reduction of the Fe_3−*x*_O_4_ NPs. The HR-TEM analysis of NPs after the Joule heating process (Supplementary Fig. 2b,c) allowed the identification of pure metallic and iron carbide phases. To quantify the effect of the Joule heating in the reduction process of the Fe-based NPs we performed an *in situ* heating of the Fe_3−*x*_O_4_-filled CNTs. The EELS spectra shown in the Supplementary Fig. 2d shows that the Fe L_3_:Fe L_2_ ratio decreases from ∼5.5 at room temperature (black line), to ∼3.7 (red line), 2.9 (blue line) after 30 min at 550 °C, and 20 min at 750 °C, respectively. Based on the good agreement between the EELS spectra of the Fe-based NPs acquired after the heating treatment under vacuum and the one acquired directly after the application of the current pulse, we have estimated that the temperature inside the CNT during the experiment is higher than 750 °C. Also, during the application of the bias voltage, most of the NPs localized inside the CNT migrate in the direction of the electron flow through its inner channel. However, several NPs do migrate through the nanotube's walls creating tunnel like structures (Supplementary Fig. 3).

### CNT to FLG nanoparticle transfer by electromigration

**Figure 3:**
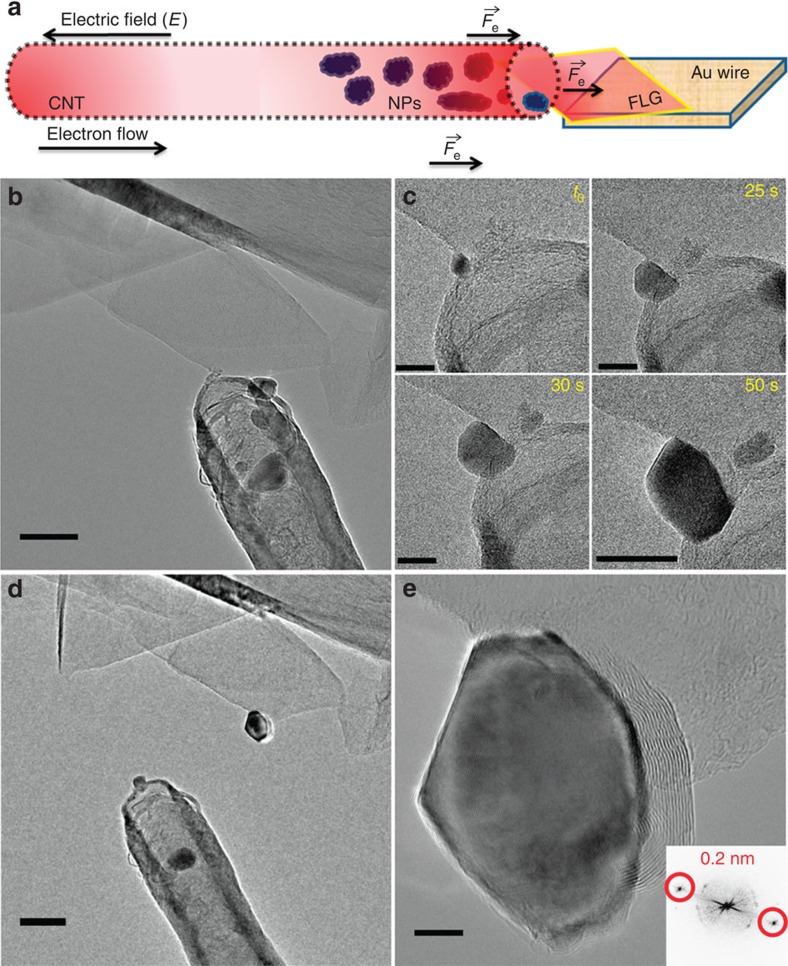
Controlled deposition of a metallic Fe nanoparticle on the edge of a FLG flake. (**a**) Schematic representation of the experiment showing the forces acting in the system on the application of a bias voltage. (**b**) The CNT/FLG system before the NP deposition. Scale bar, 50 nm. (**c**) Time series of the NP growing (time resolution 0.33 s). Scale bars, 20 nm. (**d**) The CNT/FLG system after the NP deposition on the FLG edge. Scale bar, 50 nm. (**e**) HR-TEM image of the final NP grown at the FLG edge. The FFT inset identifies a lattice spacing of about ∼0.2 nm, characteristic to *φ* and *γ* metallic Fe. Scale bar, 5 nm.

The sketch of the experimental setup used for transferring nanoparticles from the CNT to the FLG sheet is shown in Fig. 1 (for more details see Methods section). It has been previously shown that the first application of a voltage pulse structurally reorganizes the CNT through graphitization (Fig. 2b). A tube that has not been stabilized by such voltage pulses exhibits current intensity jumps as shown in Fig. 2d and is therefore not suitable for transferring nanoparticles to FLGs flakes in a controlled manner. Nevertheless, the transfer process using raw CNTs presents some interesting properties as detailed in the Supplementary Discussion. To eliminate the current intensity uncontrolled jumps during the NP's deposition, a Fe NPs/CNT system with a stabilized structure (Fig. 2b) was used. After establishing a contact between the CNT and the FLG sheet, a bias voltage is applied in such a way that the electrons do flow through the nanotube in the direction of the FLG sheet. Under such conditions the NPs in the CNT start to migrate along the tube channel towards the FLG substrate. The schematic representation of the experiment is presented in Fig. 3a. The first experiment, shown in Supplementary Movie 1, identifies nanoparticles formation on the FLG edge for a bias of about 1.5 V (which corresponds to 40–60 μA) and a bias change rate of about 15 mV s^−1^. The image of the initial CNT/FLG shown in Fig. 3b allows one to identify the presence of few nanoparticles located close to the CNT end at the FLG interface. The evolution of the system during the mass transfer from the CNT to the FLG edge versus time is presented in Fig. 3c. Supplementary Movie 1 identifies a rapid shape change of nanoparticles during the application of bias voltage. This phenomenon occurs shortly before the nanoparticle seed attends the lateral edge of the FLG flake (at *t*=0). This behaviour indicates the surface melting as event preceding the particle transfer. Right after *t*=0, the mass transfer through surface diffusion or drift of metal atoms starts and Fe atoms continue to accumulate at the FLG edge increasing the size of the new NP. At *t*=25 s a diffraction contrast is observable suggesting the presence of a crystalline aggregate acting as a seed for the subsequent growth of the NP. The nanoparticle growth progresses along both directions, parallel and perpendicular to the FLG border. It is remarkable that the nanoparticle's faceting does not vary significantly during the whole time lapse of the nanoparticle transport and growth. By resetting the bias to zero, the mass transfer instantaneously stops together with the nanoparticle evolution in terms of size and shape. Figure 3d presents the final image of the FLG border decorated with the iron nanoparticle. According to Fig. 3a–d, the NPs that were localized at the CNT's exit, at the beginning of the experiment, have ‘disappeared' almost completely, indicating their role as atoms reservoirs for the nanoparticle growth at the FLG edge. Supplementary Movie 1 shows the matter of the initial NPs slowly ripped out and further reassembled as new NPs at the FLG edge. By following the evolution of the NPs' apparent surface for the ones encapsulated in the CNT and growing on the FLG edge (Supplementary Fig. 4), one has identified a growth mechanism similar to Ostwald ripening.

Indeed, no direct migration of an entire nanoparticle from the CNT to the FLG sheet was observed. The HR-TEM image of the final nanoparticle sitting on the FLG border is shown in Fig. 3e. The Fast Fourier Transform (FFT) analysis shows that the lattice spacing of the nanoparticle is about 0.2 nm, corresponding to metallic Fe phases^23^. Additional results presented in Supplementary Fig. 5 shows a second experiment of the controlled nanoparticle deposition. The experiment (Supplementary Movie 2) shows that even when the nanoparticle is incorporating mass very rapidly within 1 s, the faceting aspect is maintained. However, by exposing the nanoparticle to high currents after the growing phase had been stopped, results in a slow but irreversible loss of the facets. The resulting nanoparticle is also a pure metallic Fe phase as for the majority of the nanoparticles transferred from the inner part of the CNT to the FLG sheet.

### Nanoparticles transfer under a high current

**Figure 4:**
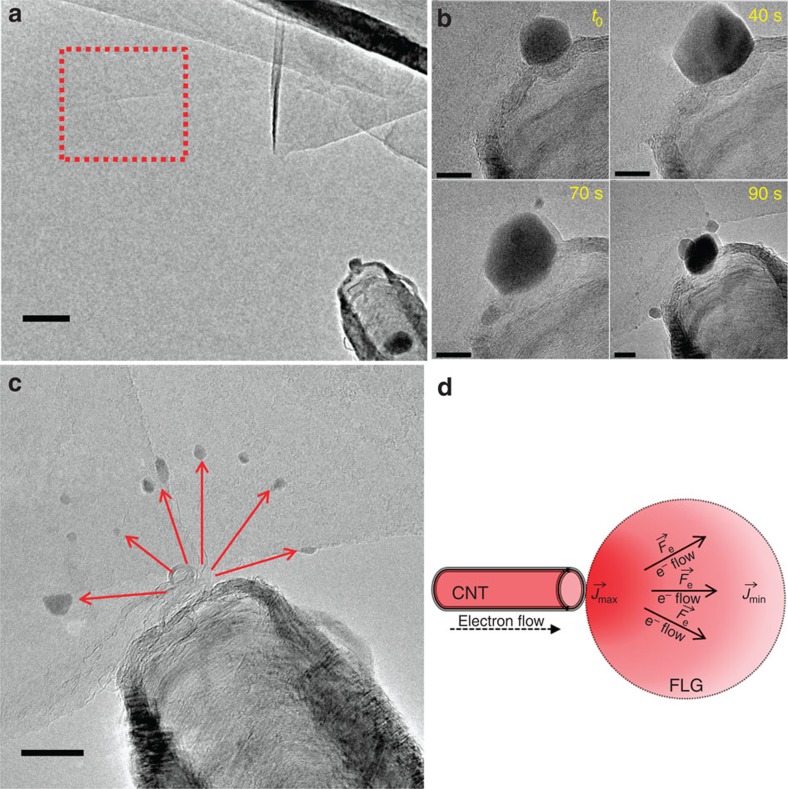
Fe-based nanoparticles radially dispersed and scattered on the FLG surface. (**a**) The CNT/FLG system before the NP transfer on the FLG surface. The exact location of the FLG area chosen for the NPs transfer is highlighted by the red square. Scale bar, 50 nm. (**b**) Time series of the uncontrolled NP growing on the upper surface of a FLG flake. Scale bars, 10 nm. (**c**) HR-TEM image of the NPs radially dispersed on the FLG surface due to the distribution of 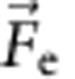 forces. Scale bar, 20 nm. (**d**) Schematic representation of the 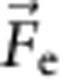 forces distribution on a FLG surface crossed by an electrical current.

The next experiment shows how the directional transfer on electromigration can be used to simultaneously deposit several nanoparticles, but with a significant loss of control in terms of final position and shape. Figure 4a presents the image of the initial system, before the nanoparticle transfer. Figure 4b displays a time series of a nanoparticle deposition when the contact between the CNT and the FLG flake is not made on the FLG border but directly on the upper surface of a FLG sheet. The nanoparticles growth process can be observed in the Supplementary Movie 3. The NPs' crystalline structure remains unchanged during the growth process even when submitted to high currents, as it is verified by the occurrence of the same well-defined crystallographic planes all along the process. By increasing the applied bias voltage up to 2.5 V the current density on the FLG surface becomes high enough (current intensity ∼110 μA) to transfer sufficient direct impulse to the metal atoms. These atoms start then to migrate and eventually recompose small nanoparticles on the FLG surface, through a migration behaviour depicted in Supplementary Movie 4. The first step consists in the formation of some NPs on the FLG border near the CNT/FLG contact interface. A few seconds later, the nanoparticles start migrating and spreading on the FLG surface along directions having a radial distribution with respect to the contact position (Fig. 4c). The predicted distribution of the electromigration force on the surface of FLG sheet is schematically represented in Fig. 4d.

### Nanoparticles transfer under inversed voltage polarity

**Figure 5:**
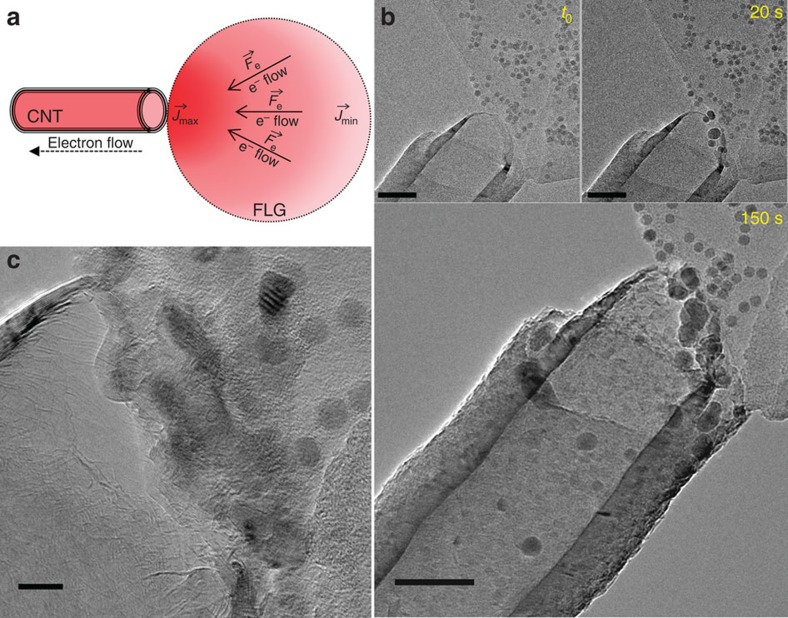
Fe-based nanoparticles transfer from a decorated FLG flake to an empty CNT. (**a**) Schematic representation of the forces distribution on a FLG surface under an electrical current (inversed polarity). (**b**) Time series of the NPs transfer inside the empty CNT. Scale bars, 50 nm. (**c**) HR-TEM image of the bulk group of NPs formed at the CNT/FLG contact after few minutes of biasing. Scale bar, 10 nm.

Removing nanoparticles deposited close to the contact point between a CNT and a FLG sheet can be simply achieved by reversing the polarity of the applied voltage (Fig. 5a). Supplementary Fig. 6 shows a time series of a nanoparticle erasure experiment. The size of the initial nanoparticle decreases slowly as its composing atoms are dragged inside the CNT and reassembled as a new nanoparticle. The situation is more problematic when the nanoparticles are not located in the immediate vicinity of the contact point. Figure 5b presents a time series of one of these experiments. The voltage is increased until the formation of small iron nanoparticles at the CNT/FLG contact is observed and then it is maintained constant. Some nanoparticles formed at the contact point start moving towards the nanotube but at the same time a bulk group is formed at the CNT/FLG interface (Fig. 5c). The nanoparticle transfer continues for a few minutes until the nanoparticles or the atomic clusters close to the contact point are exhausted. Supplementary Fig. 7 shows the evolution of the NPs' apparent surface area as deduced from the two-dimensional projections acquired before and after the transfer. We considered three groups of NPs located at different distances from the CNT/FLG contact. For these groups of NPs, the estimated amount of mass lost during the experiment increases when the distance between the NPs and the CNT/FLG contact point diminishes. Such, the NPs located in the close vicinity of the contact have lost around 13% of their mass, whereas the NPs far from the contact have lost about 10 and 8% of their mass, for distances of about 80 and 100 nm from the contact, respectively.

## Discussion

The present study shows that CNTs can be efficient nanopipelines for performing nanometric mass transfer in both directions between two conductive nanostructures, that is, here from a CNT to a graphene substrate and vice versa. By applying a bias voltage in such a way that an electron current flows through the nanotube in the direction of its contact point with the FLG, the transfer takes place from the inner part of the CNT to the FLG flakes. From a phenomenological point of view, a strong electromigration force 
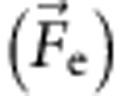
 appears along the CNT under the application of the electrical field; this force is responsible for the dragging of the iron nanoparticles from the nanotube centre to the nanotube exit end. The 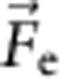 force is created by the interaction of the scattering electrons in the electric current that flows along the CNT and the metal atoms localized on the CNT inner surface; it imposes a global movement to the metal atoms along the electron flow direction^18^. Such a transfer process ‘sustained' by the electromigration force 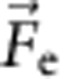, is very sensitive to the slightest uncontrolled current variation (jumps) that leads to current correlated but uncontrolled NP transfer (Supplementary Movie 5 and Supplementary Fig. 8). The main factor responsible for the current jumps is the CNT raw structure and the presence of residual amorphous carbon covering its walls. As stated previously, the amorphous to graphitic transformation of the CNT wall through Joule heating and the Fe NPs/CNT system becomes an Ohmic device when a short exposure to voltage pulses (minimum two pulses) is carried out before deposition. Nevertheless, the transfer procedure, for example, voltage interval and number of applied pulses varies from one nanotube type to another. The current flow affects as well the structure of the nanoparticles encapsulated inside the CNTs, as the initial Fe_3−*x*_O_4_ NPs are almost completely reduced to metallic and carbide phases through chemical reaction with the carbon phase of the nanotube walls. Note that the reduction of the Fe_3−*x*_O_4_ NPs is expected to take place through Joule heating, but an electrochemical reduction reaction can be also envisioned. By *in situ* heating of the Fe_3−*x*_O_4_-filled CNTs, we obtained the same EELS spectral characteristics for the O-K and Fe-L edges as the ones achieved for the same system after applying the voltage pulses only at high temperature (≥750 °C). The tunnel formation through the nanotubes' walls caused by the reaction between Fe-based nanoparticles and graphitic C shows the high chemical reactivity of the CNT walls regarding the encapsulated nanoparticles during the applied voltage pulses. Such high local reactivity can lead to the activation of a catalytic reaction between carbon and Fe, with the eventual formation of Fe carbides. To overcome these reactivity-related drawbacks, the activation procedure needs to be closely controlled. The inner surface quality (defects presence, crystallinity) before the transfer experiment is the other key parameter to account for. In this context, a careful selection of the CNTs as well the use of a well-defined activation protocols for the CNTs, in terms of voltage increasing rates, voltage intervals and number of the applied pulses will contribute to the improvement of the experiment's reproducibility. The nanoparticles transfer from the CNT channel to the FLG flakes requires a step-by-step current increase. When the 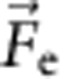 force is gaining sufficient power it starts moving atoms from the inner channel of the nanotube to the FLG flake. During the transfer process, the metal atoms are weekly linked to the graphitic layer of the inner surface of the tube which is characterized by a small amount of steps and edges due to the previous annealing of the tube, facilitating thus their diffusion and directional drift. The nanoparticle seed nucleates on the defect site localized at the closest distance to the CNT/FLG contact point. The growing behaviour of the nanoparticle is set by the seed position on the FLG, that is, on the planar surface or the edge. The nanoparticle's faceting is a continuous process occurring all along the particle growth, generally keeping a hexagonal external shape. The faceted aspect together with the phase and diffraction contrast observed in HR-TEM imaging during the growth process shows that the nanoparticle's temperature rapidly decreases in the direction perpendicular to the graphene substrate, which facilitates its continuous crystallization. The fact that the initial nanoparticle seeds act as cold-traps for the moving atoms also explains why only one single nanoparticle is formed. At the end of a transfer experiment most of the newly grown nanoparticles are in a pure metallic Fe phase. Nevertheless, Fe_*x*_C_*y*_ phases identified by lattice spacing can be found occasionally. They could be due to a local excess of the temperature favouring the formation of carbide phases. The preferential formation of metallic Fe phases on the graphene surface even if, as a result the CNT activation, the metallic and carbide iron phases are found in comparable amounts in the CNT channel, is due to the difference between the diffusion properties of Fe and C atoms under the electromigration force 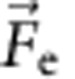 (ref. 23).

The real-time observation of the nanoparticle migration on the FLG surface shows how the CNTs can be used to simultaneously deposit more nanoparticles on the graphene substrate. The resulting radial distribution of the nanoparticles on the FLG surface can be associated with the radial distribution of the current lines away from the CNT/FLG contact point. The migration is promoted by an Ostwald ripening-like mechanism. In the opposite configuration, when the current polarity is reversed, an empty CNT can be used to remove nanoparticles from a decorated FLG sheet. In this case the 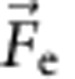 force transport the metal atoms inside the nanotube where they will recompose forming new nanoparticles. However, a higher bias voltage (and thus a stronger 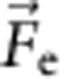) is needed for their transfer, as they should be generally collected from regions with low current density, far away from the CNT/FLG contact point. Therefore, keeping the system at a constant voltage, but below its breaking point will allow only the transfer of the nanoparticles located close the CNT/FLG contact.

In conclusion, using a combined TEM and STM technique associated to electron spectroscopy, we were able to define conditions under which a reliable electrically controlled transfer of NPs from the inner part of a CNT onto a FLG sheet can be performed. From a general point of view, the mass transfer requires four individual steps: the annealing of the tube, the chemical transformation or the reduction of the original particles, the particle transfer through the tube and the dispensing of the metal atoms on the graphene sheet. The erasing of already deposited NPs can also be achieved by reversing the applied electrical voltage. The printing process described in this work mimics the process used in a ‘dye sublimation printer' and we therefore consider that this study is the proof-of-concept for the design of printing devices at the nanoscale. However, future studies should provide a better control of the nanoprinting process. The tip could be automatically moved according to a geometric pattern allowing metal deposition in both continuous and discrete modes, as a function of the applied voltage.

## Methods

### Synthesis of Fe_3_O_4_-filled CNTs and FLG sheets

The filling of commercial multi-wall CNTs (MW-CNT) with Fe_3−*x*_O_4_ NPs was performed using the thermal decomposition of iron stearate^24^, procedure that was used as well for the deposition of Fe_3−*x*_O_4_ NPs on the FLG^25^. The graphene samples were synthetized from expanded graphite by surfactant-assisted high power ultra-sonication and are characterized by large surface areas (a few μm^2^) and few layers in thickness. Meanwhile, an electrochemical etching method was used to prepare Au tips^26^, which were used as contact bridges. For the manufacturing of both Au wire electrodes and tips, we have employed commercial 3 mm diameter wires and the tip diameter obtained was around 50 nm. After their synthesis has been performed, the Fe_3−*x*_O_4_-filled CNTs and graphene samples were dispersed in ethanol and subsequently deposited on the movable Au tip and the fixed Au wire, respectively.

### Experimental TEM–STM setup

The two Au contacts (the tip and the Au wire) were fixed in the nanofactory TEM–STM holder, which permits a sub-nanometre precision in sample positioning during the experiment. The schematic drawing of the experimental setup is shown in Fig. 1. Characteristic *I*(*V*) curves were recorded over the voltage interval 0–3 V with a scanning time of 100 ms. The real-time observation (0.33 s time resolution) and the HR-TEM imaging were performed using a JEOL 2100F microscope with a high voltage of 200 kV, equipped with a high-resolution objective lens pole piece. Under this configuration, images with a spatial resolution of ∼0.2 nm were recorded. EELS spectra were recorded using a GATAN Tridiem imaging filter in the parallel mode with a 2 mm aperture. For the experiments needing an *in situ* heating, a few drops of Fe_3−*x*_O_4_ NPs-filled CNTs ethanol solution were deposited on a holey carbon film, which was later mounted in a Gatan double tilt heating holder. The temperature ramp speed was about 0.5 °C s^−1^.

## Additional information

**How to cite this article:** Melinte, G. *et al.* Towards nanoprinting with metals on graphene. *Nat. Commun.* 6:8071 doi: 10.1038/ncomms9071 (2015).

## Supplementary Material

Supplementary Information Supplementary Figures 1-8 and Supplementary Discussion.

Supplementary Movie 1 The controlled transfer of a metallic Fe nanoparticle from a CNT to the FLG flake's edge (3x real time). 23s: surface melting of the encapsulated NPs; 33s: beginning of the NP transfer process.

Supplementary Movie 2 The controlled transfer of a metallic Fe nanoparticle from a CNT to the FLG flakes upper surface (2x real time).

Supplementary Movie 3 HR-TEM imaging of an uncontrolled nanoparticle growing on the FLG flake surface (3x real time).

Supplementary Movie 4 Real time observation of the nanoparticles migrating on the FLG flake's surface (3x real time).

Supplementary Movie 5 Uncontrolled nanoparticle transfer using a raw Fe3-xO4 filled CNT (3x real time).

## References

[b1] Geim A. K. Graphene: status and prospects. Science 324, 1530–1534 (2009). 19541989 10.1126/science.1158877

[b2] Baughman R. H., Zakhidov A. A. & de Heer W. A. Carbon nanotubes-the route toward applications. Science 297, 787–792 (2002). 12161643 10.1126/science.1060928

[b3] Close G. F., Yasuda S., Paul B., Fujita S. & Wong H.-S. P. A 1 GHz integrated circuit with carbon nanotube interconnects and silicon transistors. Nano Lett. 8, 706–709 (2008). 18269256 10.1021/nl0730965

[b4] Markus Löffler U. W. Current-induced mass transport in filled multiwalled carbon nanotubes. Adv. Mater. 23, 541–544 (2011). 21254260 10.1002/adma.201002247

[b5] Coh S., Gannett W., Zettl A., Cohen M. L. & Louie S. G. Surface atom motion to move iron nanocrystals through constrictions in carbon nanotubes under the action of an electric current. Phys. Rev. Lett. 110, 185901 (2013). 23683222 10.1103/PhysRevLett.110.185901

[b6] Golberg D. *et al.* Copper-filled carbon nanotubes: rheostatlike behavior and femtogram copper mass transport. Adv. Mater. 19, 1937–1942 (2007).

[b7] Sun L. *et al.* Carbon nanotubes as high-pressure cylinders and nanoextruders. Science 312, 1199–1202 (2006). 16728637 10.1126/science.1124594

[b8] Sutter P. W. & Sutter E. A. Dispensing and surface-induced crystallization of zeptolitre liquid metal-alloy drops. Nat. Mater. 6, 363–366 (2007). 17435761 10.1038/nmat1894

[b9] Dorozhkin P. S. *et al.* A liquid-Ga-filled carbon nanotube: a miniaturized temperature sensor and electrical switch. Small 1, 1088–1093 (2005). 17193401 10.1002/smll.200500154

[b10] Sun M. & Gao Y. Electrically driven gallium movement in carbon nanotubes. Nanotechnology 23, 065704 (2012). 22248658 10.1088/0957-4484/23/6/065704

[b11] Scheuermann G. M., Rumi L., Steurer P., Bannwarth W. & Mülhaupt R. Palladium nanoparticles on graphite oxide and its functionalized graphene derivatives as highly active catalysts for the Suzuki-Miyaura coupling reaction. J. Am. Chem. Soc. 131, 8262–8270 (2009). 19469566 10.1021/ja901105a

[b12] Xu C., Wang X. & Zhu J. Graphene-metal particle nanocomposites. J. Phys. Chem. C 112, 19841–19845 (2008).

[b13] Wu Z.-S. *et al.* Graphene anchored with Co_3_O_4_ nanoparticles as anode of lithium ion batteries with enhanced reversible capacity and cyclic performance. ACS Nano 4, 3187–3194 (2010). 20455594 10.1021/nn100740x

[b14] Huang X. *et al.* Graphene-based materials: synthesis, characterization, properties, and applications. Small 7, 1876–1902 (2011). 21630440 10.1002/smll.201002009

[b15] Cao A. *et al.* A facile one-step method to produce graphene-CdS quantum dot nanocomposites as promising optoelectronic materials. Adv. Mater. 22, 103–106 (2010). 20217706 10.1002/adma.200901920

[b16] Dong L., Tao X., Zhang L., Zhang X. & Nelson B. J. Nanorobotic spot welding: controlled metal deposition with attogram precision from copper-filled carbon nanotubes. Nano Lett. 7, 58–63 (2007). 17212440 10.1021/nl061980+

[b17] Zou R. *et al.* Melting of metallic electrodes and their flowing through a carbon nanotube channel within a device. Adv. Mater. 25, 2693–2699 (2013). 23559074 10.1002/adma.201300257

[b18] Král P. & Wang B. Material drag phenomena in nanotubes. Chem. Rev. 113, 3372–3390 (2013). 23410181 10.1021/cr200244h

[b19] Regan B. C., Aloni S., Ritchie R. O., Dahmen U. & Zettl A. Carbon nanotubes as nanoscale mass conveyors. Nature 428, 924–927 (2004). 15118721 10.1038/nature02496

[b20] Svensson K., Olin H. & Olsson E. Nanopipettes for metal transport. Phys. Rev. Lett. 93, 145901 (2004). 15524812 10.1103/PhysRevLett.93.145901

[b21] Svensson K., Jompol Y., Olin H. & Olsson E. Compact design of a transmission electron microscope-scanning tunneling microscope holder with three-dimensional coarse motion. Rev. Sci. Instrum. 74, 4945–4947 (2003).

[b22] Huang J.-Y., Ding F., Jiao K. & Yakobson B. I. Self-templated growth of carbon-nanotube walls at high temperatures. Small 3, 1735–1739 (2007). 17763513 10.1002/smll.200700105

[b23] La Torre A. *et al.* Formation and characterization of carbon-metal nano-contacts. Carbon 77, 906–911 (2014).

[b24] Baaziz W. *et al.* Carbon nanotube channels selectively filled with monodispersed Fe_3−*x*_O_4_ nanoparticles. J. Mater. Chem. A 1, 13853–13861 (2013).

[b25] Baaziz W. *et al.* Few layer graphene decorated with homogeneous magnetic Fe_3_O_4_ nanoparticles with tunable covering densities. J. Mater. Chem. A 2, 2690–2700 (2014).

[b26] Ren B., Picardi G. & Pettinger B. Preparation of gold tips suitable for tip-enhanced Raman spectroscopy and light emission by electrochemical etching. Rev. Sci. Instrum. 75, 837–841 (2004).

