# Supplementary material for: Towards nanoprinting with metals on graphene
Source: Nat Commun. 2015 Aug 28;6:8071. doi: 10.1038/ncomms9071 (PMC4560798; doi:10.1038/ncomms9071)
Supplement: Supplementary Information — Supplementary Figures 1-8 and Supplementary Discussion. [file ncomms9071-s1.pdf]

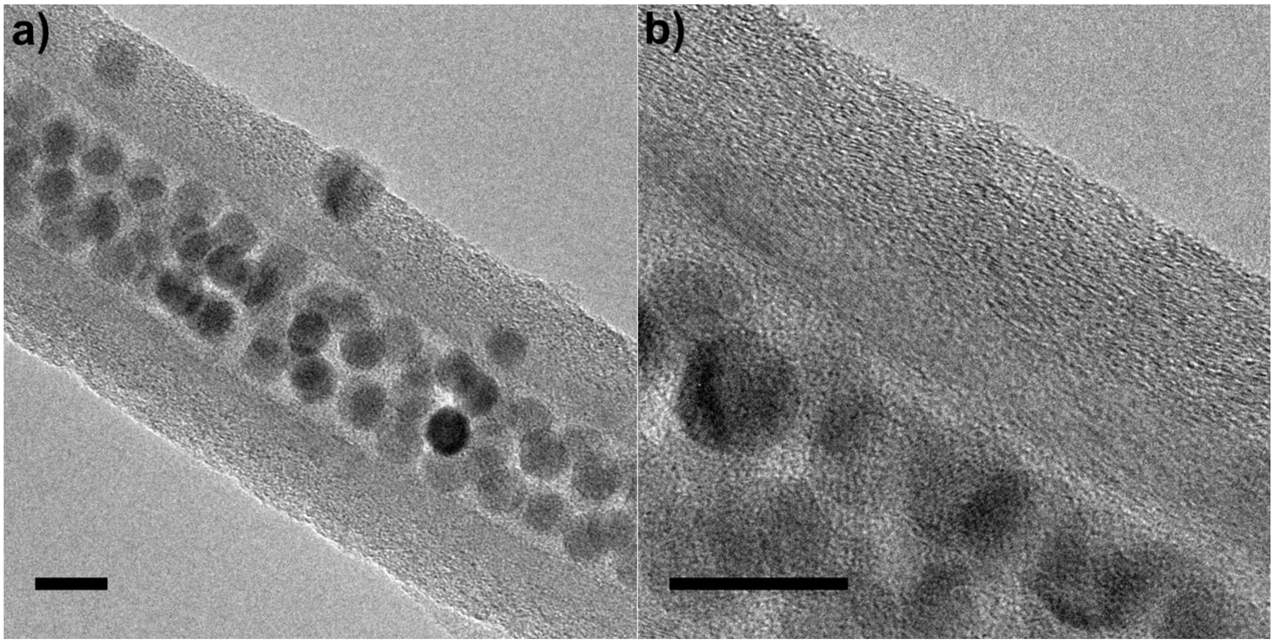


**Supplementary Figure 1.** TEM images of the initial $Fe_{3-x}O_4$ NPs filled CNTs. TEM (a) and HR-TEM (b) images showing the structure of their thick walls which contain a large amount of amorphous carbon. Scale bars 20 nm.

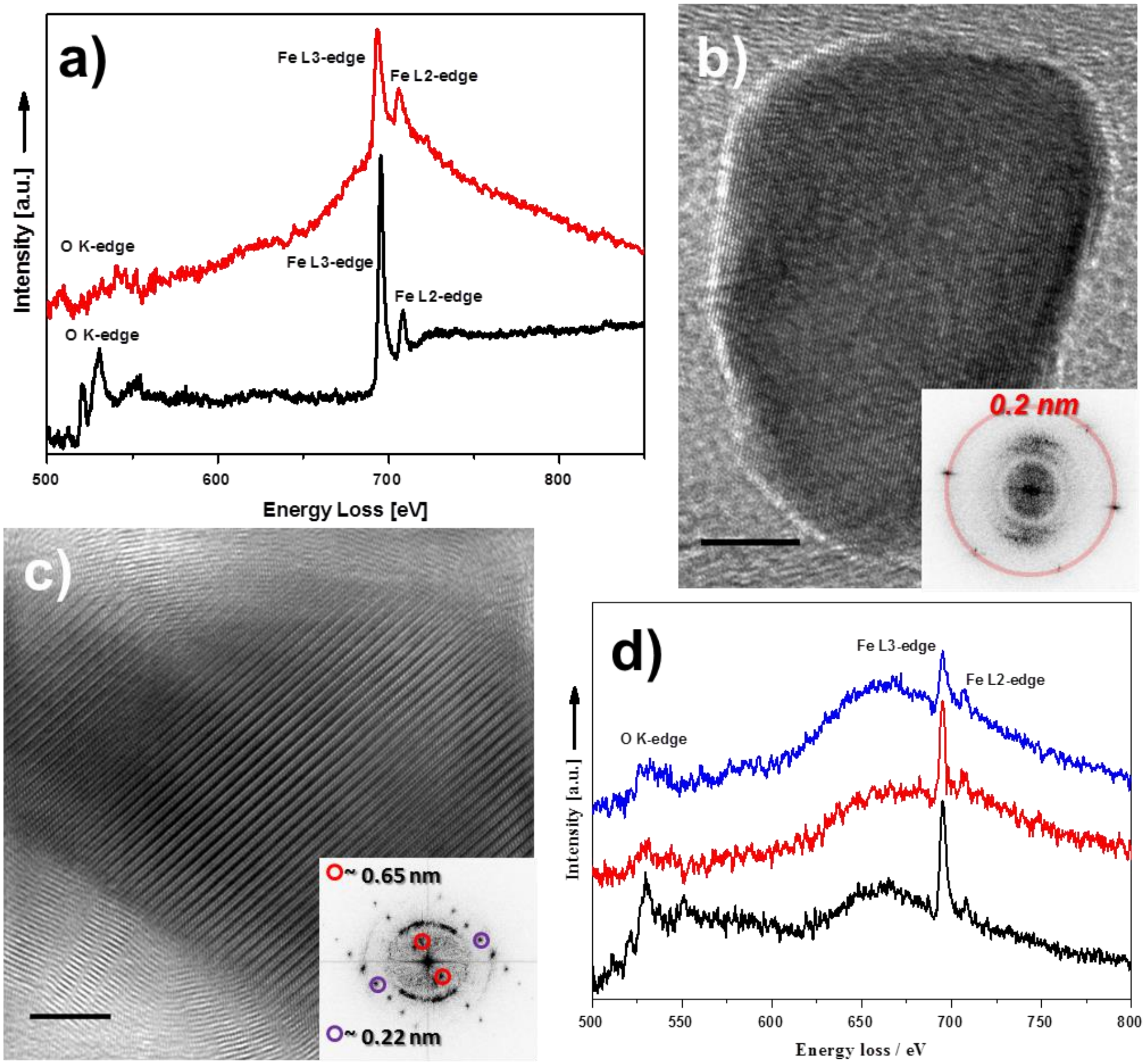


**Supplementary Figure 2.** EELS and HR-TEM investigations of the nanoparticles obtained during the Joule activation of the CNTs.

a) EELS spectra of the Fe based NPs filled CNT before (black line/ $Fe_{3-x}O_4$) and after (red line/metallic Fe and $Fe_xC_y$ phases) the application of the voltage pulse.

b) HR-TEM image of a metallic Fe nanoparticle formed during the application of a voltage pulse on the $Fe_{3-x}O_4$ filled CNT. The FFT inset shows a lattice spacing of ~0.2 nm characteristic to both ϕ and γ Fe phases. Scale bar 5 nm.

c) HR-TEM image of a $Fe_xC_y$ phase resulting from the Joule annealing of the initial $Fe_{3-x}O_4$ filled CNT. The FFT inset shows a large lattice spacing characteristic to complex iron carbide phases. Scale bar 5 nm.

d) EEL spectra of the Fe based NPs filled CNTs during in-situ heating black line – room temperature, red line – 550 °C for 30 min, blue line - 750 °C for 20 min.

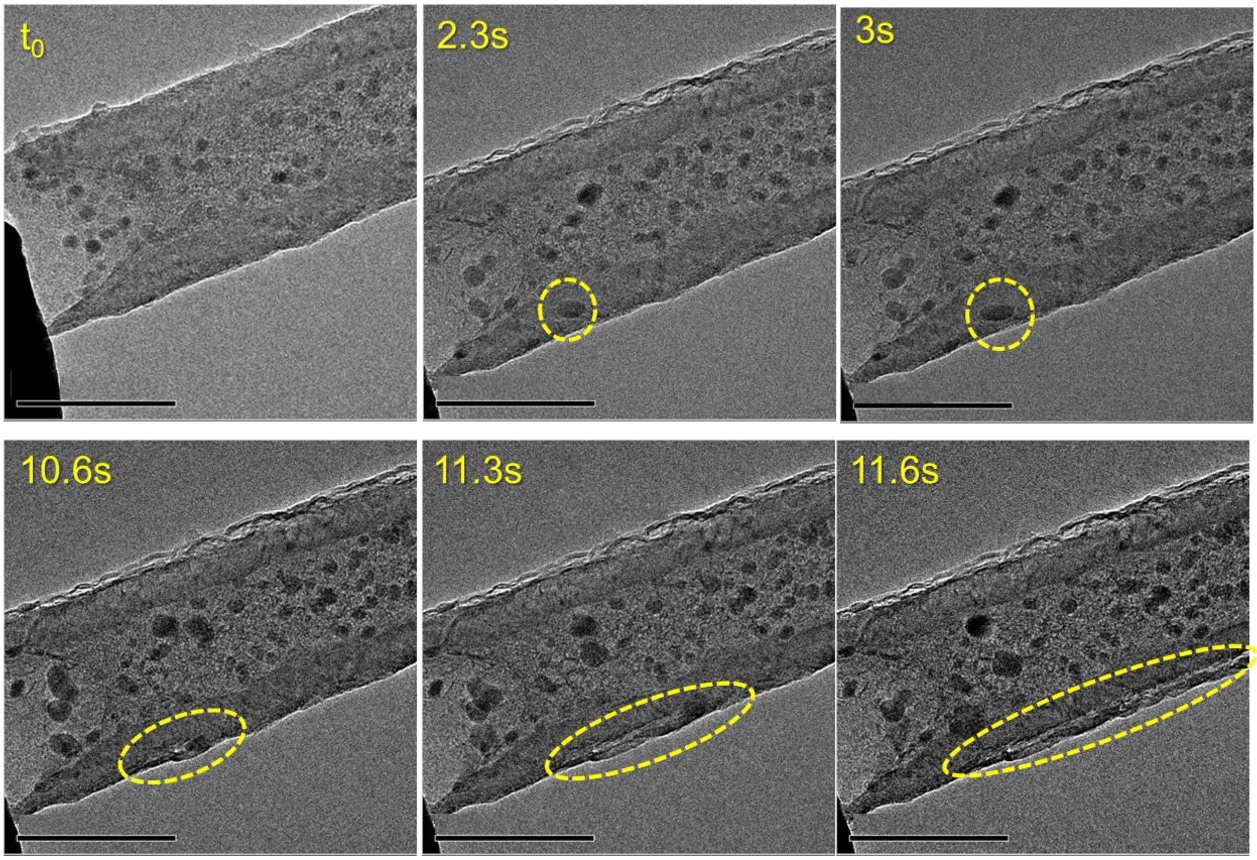


**Supplementary Figure 3.** Time series of a Fe based NP digging a tunnel through the nanotube wall under the impulse from the electromigration force, $\overrightarrow{F_e}$. The total experiment time was under 12 s. Scale bars 100 nm.

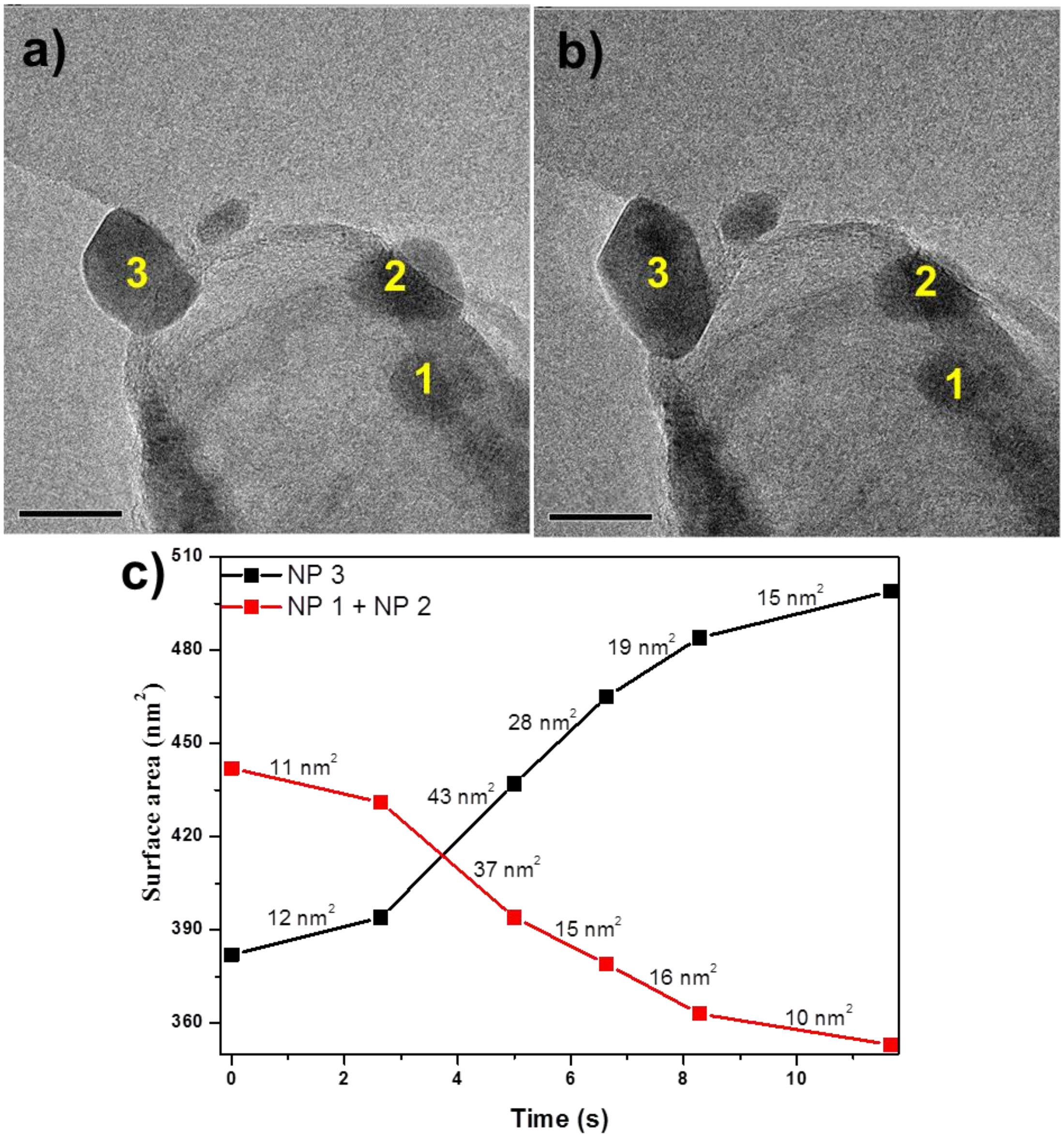


**Supplementary Figure 4.** The size evolution of the nanoparticles during a controlled deposition experiment. TEM images extracted from the Supplementary Movie 1 corresponding to the t0 (a) and 11.66 s (b) positions. Scale bars 20 nm. c) The evolution of the apparent surface area of the NPs encapsulated in the CNT (summed area of NPs 1 and 2) and of the one deposited on the FLG edge (NP 3). The numerical values evaluated for each time segment show the exact area loss (red segment) and gain (black segment) for the corresponding interval.

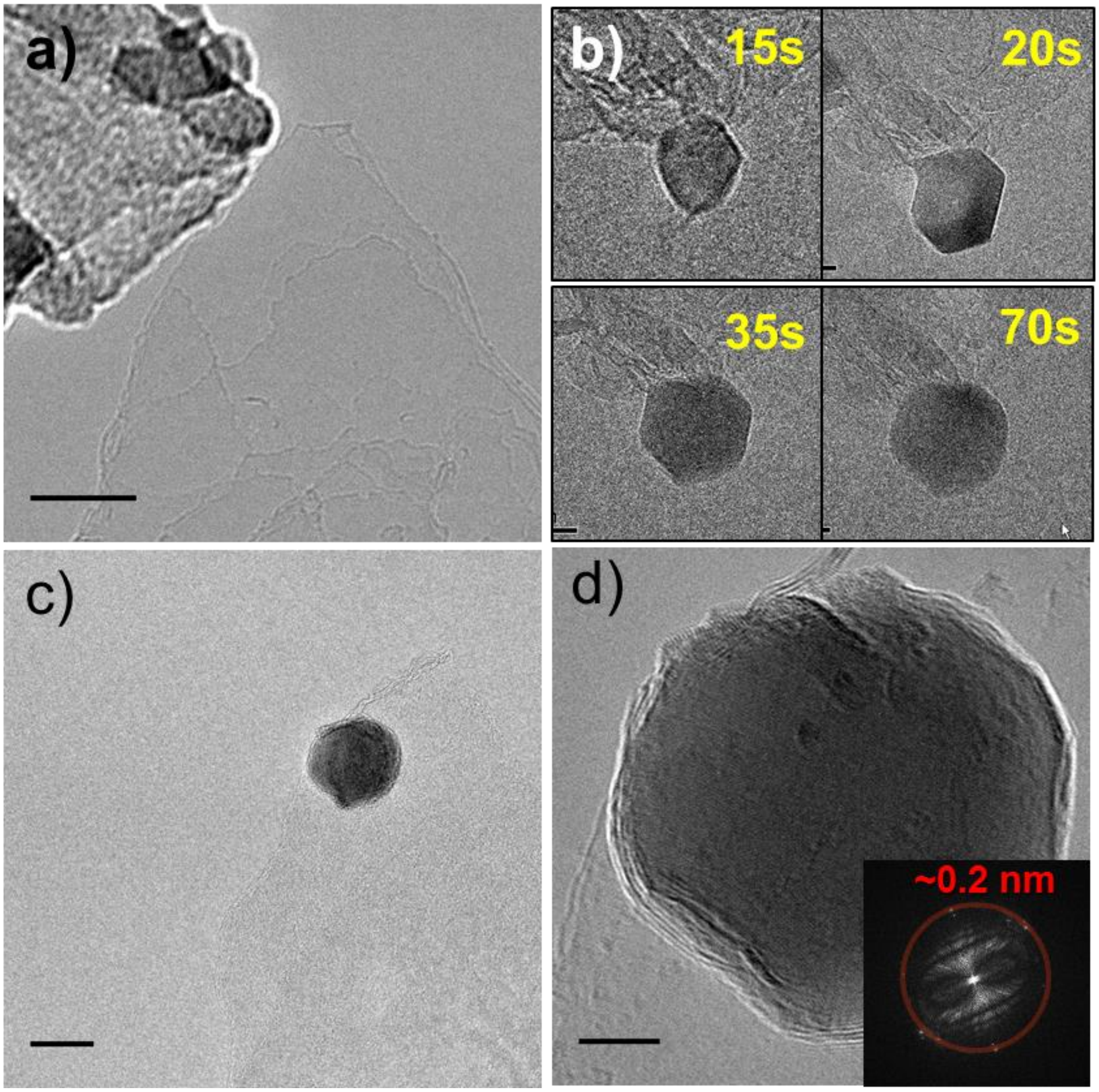


**Supplementary Figure 5.** The controlled deposition of a metallic Fe nanoparticle on the upper surface of a FLG sheet. a) The CNT/FLG system before the application of the driving voltage; Scale bar 20 nm. b) A time series of the NP growing (time resolution 0.33s); c) The FLG system after the NP deposition on its surface; Scale bar 20 nm. d) HR-TEM image of the final metallic Fe NP deposited on the FLG surface. The FFT shows a lattice spacing of about ~0.2 nm, characteristic for $\phi$ and $\gamma$ metallic Fe. Scale bar 5 nm.

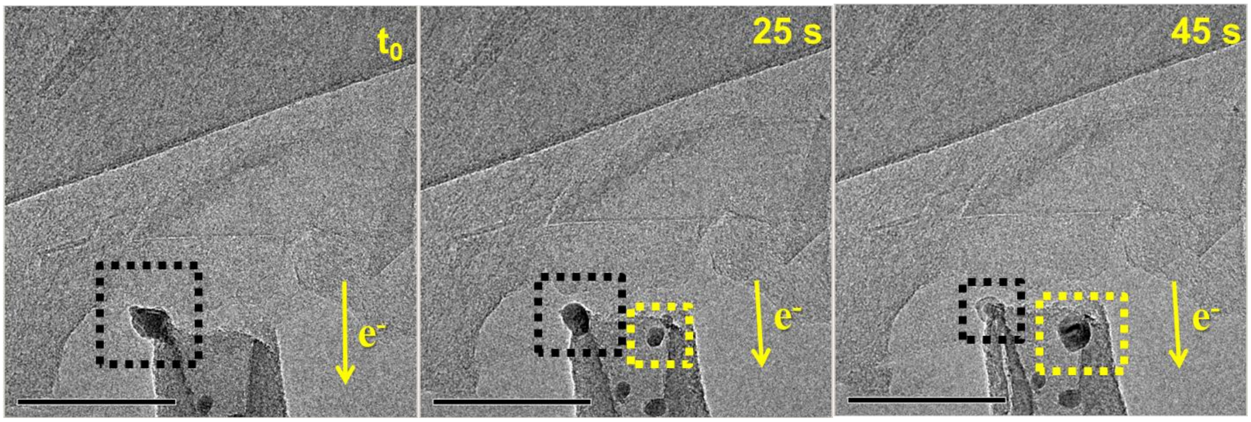

**Supplementary Figure 6.** Time series of a nanoparticle removal experiment from a position close to the CNT/FLG interface. The positions of the initial and of the reassembled nanoparticle are highlighted by black and yellow squares, respectively. Scale bars 100 nm.

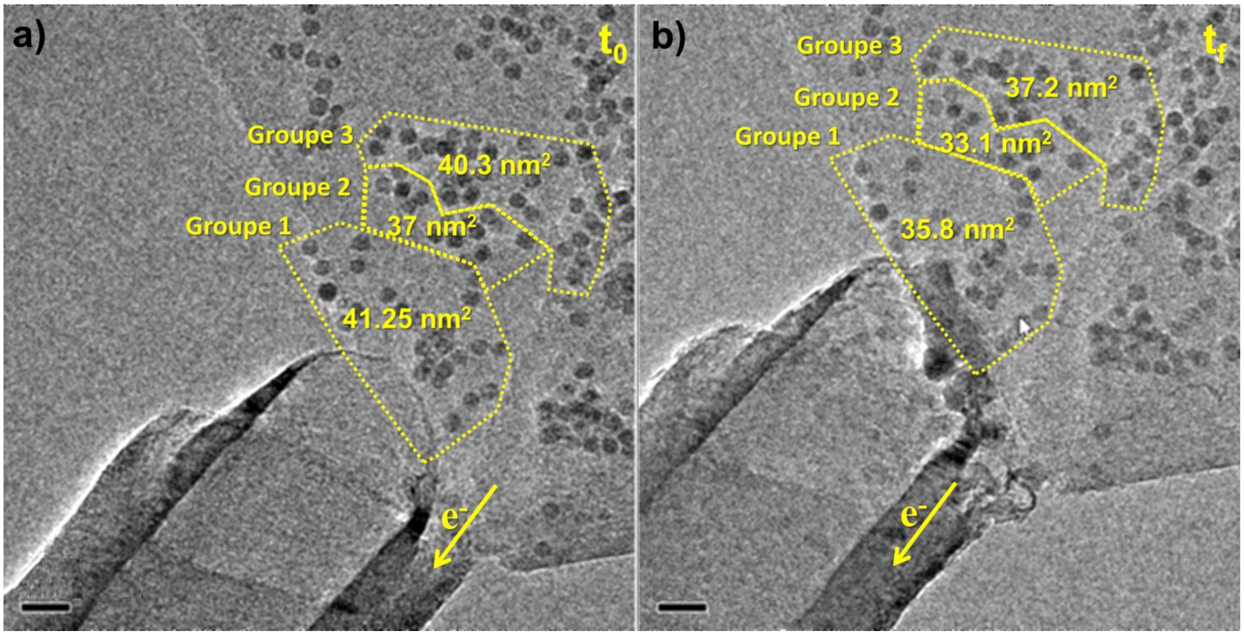


**Supplementary Figure 7.** The evolution of the nanoparticle apparent surface during the erasing experiment shown in Figure 5. a) TEM image of the initial system. The nanoparticles were divided into three groups as a function of their position with respect to the CNT/FLG contact (highlighted in yellow). The numerical values represent the mean surface area for the nanoparticles from each group. b) TEM image of the system after the erasing experiment. The differences between the mean apparent surfaces of the NPs from each group before and after the erasing experiment are: ~5.5 nm$^2$ (13%) (group 1), ~3.9 nm$^2$ (10%)(group 2), and 3.1 nm$^2$ (8%) (group 3). Scale bars 20 nm.

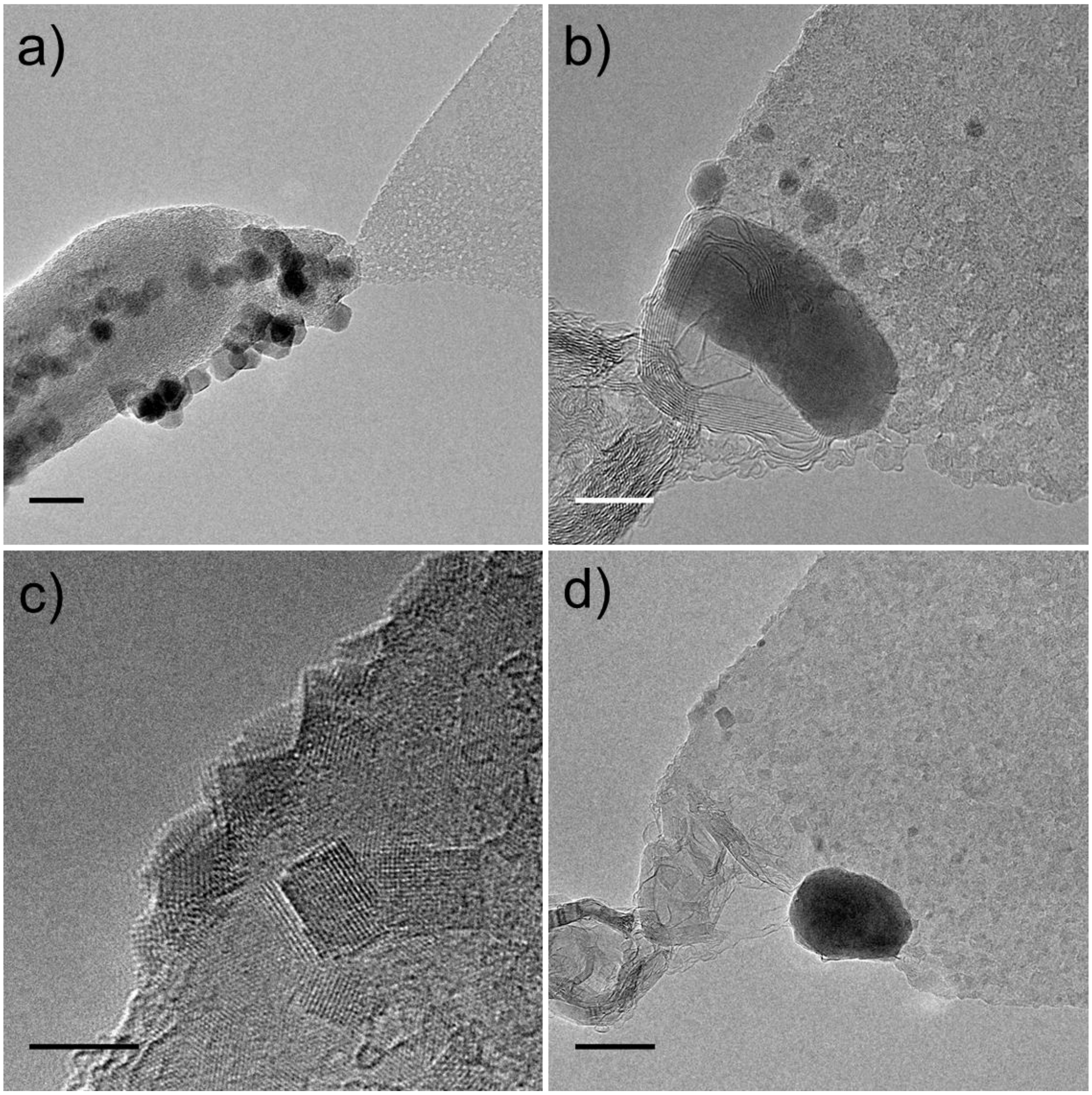


**Supplementary Figure 8.** The uncontrolled transfer of Fe based nanoparticles on the upper surface of a FLG sheet. a) The CNT/FLG system before the NP deposition; Scale bar 20 nm. b) The graphitized CNT/FLG contact due to the Fe NP action at high temperature; Scale bar 10 nm. c) Small Fe clusters decorating the surface of the FLG; Scale bar 5 nm. d) The final state of the FLG flake decorated with iron NPs in a uncontrolled way. Scale bar 20 nm. These mages correspond to the experiment shown in Supplementary Movie 5.

**Supplementary Discussion**

The direct use of a raw nanotube leads to an uncontrolled migration of the nanoparticles under the application of a bias voltage. This results from the structural reorganization undergone by the CNT during the first application of the bias. Supplementary Movie 5 presents one of these experiments in high-resolution. The initial stage of the CNT/FLG system is presented in Supplementary Fig. 8a. Some of the $Fe_{3-x}O_4$ NPs are attached to the nanotube external surface. After the nanotube was connected to the FLG sheet the bias is stepped up by about 15mV/s. The first observation of the nanoparticles' melting takes place after 72s at a current intensity of ~51μA. At first, the melting appears only for two small nanoparticles located in the close vicinity of the CNT/FLG contact. The fact that the melting starts with these particles shows that the contact resistance between the CNT and FLG is slightly higher than the overall resistance of the CNT and this generates a higher Joule heating in the contact vicinity. Then the bias is kept at the same value for ~12s and no major modification occurs showing that the system's temperature rapidly reaches a uniform distribution. When the bias starts increasing again, more NPs do melt close to the CNT/FLG contact point (56μA) followed by a rapid "backward" moving of the NPs against the direction of the $\overrightarrow{F_e}$ (88s). The "backward" migration of the nanoparticles sitting at the end of a CNT close to the contact with the FLG sheet is a common phenomenon when the current is instantly jumping to higher values. This is due to a temperature gradient arising from the higher temperature reached at the contact point between the CNT and the FLG that forces the melted nanoparticles to move to a colder region inside the CNT (thermomigration). The thermomigration force ($\overrightarrow{F_t}$) only appears when the current increases in an uncontrolled way and rapidly stops due to the stabilization of the system's temperature. The nanoparticle melting first appears inside the nanotube while the nanoparticles sitting on the tube walls remain stable for a longer time. Around t=100s at a current intensity of 58μA nanoparticles do appear on the FLG surface. The iron continues to accumulate on the FLG surface in an uncontrolled way and around t=125s (78μA), due to the high temperature reached, the nanoparticle does graphitize the CNT/FLG contact (Supplementary Fig. 8b). However the contact graphitization does not induce an increase of the current flowing through the system. When the bias is further increased the nanoparticles will start moving on the FLG surface continuing to graphitize it. At the end of the experiment, after reaching a maximum current intensity of 220μA, the FLG surface is decorated with a 30nm in

diameter metallic Fe nanoparticle and small iron clusters ripped off from the large nanoparticle (Supplementary Fig. 8c and 8d).
